# Cell Death and Exosomes Regulation After Myocardial Infarction and Ischemia-Reperfusion

**DOI:** 10.3389/fcell.2021.673677

**Published:** 2021-06-09

**Authors:** Xun Wu, Chukwuemeka Daniel Iroegbu, Jun Peng, Jianjun Guo, Jinfu Yang, Chengming Fan

**Affiliations:** ^1^Department of Cardiovascular Surgery, The Second Xiangya Hospital, Central South University, Changsha, China; ^2^Hunan Provincial Key Laboratory of Cardiovascular Research, Changsha, China; ^3^Hunan Fangsheng Pharmaceutical Co., Ltd., Changsha, China

**Keywords:** myocardial infarction, apoptosis, autophagy-dependent death, pyroptosis, ferroptosis, exosomes, microRNA, myocardial protection

## Abstract

Cardiovascular disease (CVD) is the leading cause of death in the global population, accounting for about one-third of all deaths each year. Notably, with CVDs, myocardial damages result from myocardial infarction (MI) or cardiac arrhythmias caused by interrupted blood flow. Significantly, in the process of MI or myocardial ischemic-reperfusion (I/R) injury, both regulated and non-regulated cell death methods are involved. The critical factor for patients’ prognosis is the infarct area’s size, which determines the myocardial cells’ survival. Cell therapy for MI has been a research hotspot in recent years; however, exosomes secreted by cells have attracted much attention following shortcomings concerning immunogens. Exosomes are extracellular vesicles containing several biologically active substances such as lipids, nucleic acids, and proteins. New evidence suggests that exosomes play a crucial role in regulating cell death after MI as exosomes of various stem cells can participate in the cell damage process after MI. Hence, in the review herein, we focused on introducing various cell-derived exosomes to reduce cell death after MI by regulating the cell death pathway to understand myocardial repair mechanisms better and provide a reference for clinical treatment.

## Introduction

Despite the considerable improvements in healthcare worldwide, acute myocardial infarction (MI) has long been the primary cause of death from coronary heart diseases (Roth et al., 2017). Endogenous cardiomyocytes have limited renewal potentials following MI, which leads to the irreversible loss of a significant number of cardiomyocytes, left ventricular remodeling, and progressive heart failure (Buja and Vela, 2008). Therefore, rescuing damaged cardiomyocytes becomes a potential strategy to preventing myocardial dysfunction and heart failure after MI.

Exosomes are small-membrane fragments with a diameter of 30–150 nm, secreted by almost all mammalian cells. Exosomes arise from a multivesicular endosomal pathway by fusion of multivesicular bodies (MVBs) with the plasma membrane, resulting in the release of vesicles as exosomes into the extracellular space. The generation of exosomes in MVBs can be divided into endosomal independent sorting complexes required for transport (ESCRT) and ESCRT-dependent mechanisms (Hurley, 2008; Stuffers et al., 2009). For example, cytoplasmic proteins’ isolation into exosomes may result from co-classification with other proteins, for which heat shock protein70 (HSP70) plays an essential role (Théry et al., 2001). RNAs may also be classified into exosomes with complex mechanisms, including binding ESCRT-II subcomplex to RNA (Irion and St Johnston, 2007) and the sumoylation of hnRNPA2B1 (Villarroya-Beltri et al., 2013). The secretion of exosomes also requires MVBs to fuse with the plasma membrane. The process may be mediated by SNARE proteins and members of the synaptotagmin family (Jahn and Scheller, 2006). Exosomes are characterized by lipid bilayers, annexin, GTPase, Rab, transmembrane, and non-membrane-bound proteins, flotillin proteins, and ESCRT components, which include Alix, HSPs, Tsg101, integrins, and transmembrane proteins (CD63, CD81, and CD82). Exosomes also express secretory cell markers. Besides, exosomes are also rich in RNA from different sources (Théry et al., 2002).

The study herein reviews the potential mechanism of cell-derived exosomes in repairing damaged cardiomyocytes and provides a reference point for future clinical treatment of patients with cardiac ischemic diseases. It should be noted that that exosome is a two-edged sword during medical therapy. Following MI, exosomes may simultaneously enhance blood vessels’ regeneration and damaged myocardium in the area around the infarcted site. The review aims to illustrate the therapeutic effect (anti-cell death) and potential molecular mechanism after myocardial ischemia.

## Types of Cardiomyocyte Death and the Potential Mechanisms and Key Mediators of Cell Death in Cardiac Diseases

Following MI, cardiac I/R injury, heart failure, and other heart diseases, cell death can occur in regulated and non-regulated forms (Moe and Marín-García, 2016; Shekhar et al., 2018). Significantly, MI is caused by acute or chronic tissue deficiency of oxygen, nutrients, and growth factors. Although related imbalances following a series of MI could arise from several pathological cascades, the most common cause is acute or prolonged ischemia, with the rupture of coronary atherosclerotic plaques being the primary cause (Libby and Pasterkamp, 2015). In the 1990s, the implantation of thrombolysis or stents was considered an effective way to reduce the size of MI (Nabel and Braunwald, 2012). Although the potential risk of perfusion is still questionable after a few years, it is clear that the perfusion process will cause cell death via oxidative stress, calcium overload, and inflammation (Yellon and Hausenloy, 2007). Notwithstanding, it is difficult to assess the significant role ischemic duration and reperfusion play in the damaged myocytes. In most cases, genetic and pharmacological inhibition of cell death signaling reduces cardiomyocyte death and infarct size only in the context of myocardial ischemia-reperfusion. However, such changes are uncommon in permanent ischemia models. Remarkably, the primary reason will be the change from initially regulated cell death to unregulated cell death if the death stimulus persists (Del Re et al., 2019). Notably, extensive studies have been conducted on MI; it is still unclear which death process is dominant.

Morphological criteria consider that cell necrosis is the primary mode of myocardial cell death after MI. In contrast, apoptosis is considered to be the first regulated cell death process. Several studies have shown that the inhibition of apoptosis receptors and the reduction of mitochondrial apoptosis reduce apoptosis of cardiomyocytes and reduce the size of MII (Jeremias et al., 2000; Chen et al., 2001). Apart from apoptosis, studies show that necroptosis (Newton et al., 2016), pyroptosis (Kawaguchi et al., 2011), ferroptosis (Fang et al., 2019), parthanatos (Yang et al., 2000), autophagy-dependent cell death (Matsui et al., 2007), and mitochondrial-dependent necrosis (Baines et al., 2005) are all involved in cell death during MI. Thus, intervention is crucial as it can significantly mitigate the number of dead cardiomyocytes in peri-infarcted areas. These studies indicated that cardiomyocytes’ death during MI was not based on a specific individual death pathway.

## Apoptosis

Apoptosis, a kind of programmed death, is a different way of cell death from necrosis Apoptotic cells undergo structural changes, including cell shrinkage, nuclear pyknosis, and fragmentation (Edinger and Thompson, 2004). Studies have shown that cardiomyocyte apoptosis begins after prolonged myocardial ischemia or reperfusion after short-term ischemia (Kajstura et al., 1996). Activation of pro-apoptotic factors and caspase can be detected in the absence of DNA breakage during ischemia, followed by a substantial increase during reperfusion, suggesting that the apoptotic cascade begins during ischemia but fully implemented during reperfusion (Lazou et al., 2006). Clinical studies have confirmed apoptotic cardiomyocytes in the marginal regions of an infarcted area within hours to days after acute infarction (Saraste et al., 1997).

Apoptosis is mediated by two well-defined pathways, extrinsic and intrinsic, both of which are activated in cardiomyocytes under pathophysiological conditions (Youle and Strasser, 2008). In the exogenous pathway, cell death is induced by the activation of death domain receptors on the cell membrane. It is triggered by Fas ligand or tumor necrosis factor (TNF)-α (Ashkenazi and Dixit, 1998). Increased expression of Fas and TNF-α is associated with increased apoptosis in cardiomyocytes in a model of I/R heart disease. Both Fas and TNF receptors have an intracellular death domain that recruits and activates caspase-8 in the cell membrane. The recruitment of caspase-8 then activates downstream caspases (including caspase-3) (Torre-Amione et al., 1996). Intrinsic pathways are activated by intracellular stress signals, such as hypoxia, oxidative stress, and DNA damage (Edinger and Thompson, 2004). The intrinsic pathway is regulated by the Bcl-2 protein family (Adams and Cory, 1998). Oligomerization of Bax and Bak in the Bcl-2 family results in pores formation, leading to the release of cytotoxic proteins from the mitochondria such as cytochrome C. Cytochrome C then activate procaspase-9, and the activated caspase-9 then cleaves and activates caspase-3 and caspase-7 (Adams and Cory, 1998).

## Autophagy

Autophagy is an essential metabolic process where aging or damaged proteins and organelles are broken down into amino acids and fatty acids for energy generation and recycling (Cǎtanǎ et al., 2018). These metabolic processes are activated during nutrient deficiency or metabolic stress to maintain tissue function and dynamic balance (Dong et al., 2018). It has been proved that autophagy is essential for maintaining normal heart function (Zhang Q. Y. et al., 2018). Amongst scholars, it is believed that autophagy is an essential lysosome-dependent catabolic mechanism (Hewitt and Korolchuk, 2017). According to lysosomes’ transport mode and physiological function, autophagy is primarily divided into three types: macroautophagy, microautophagy, and chaperone-mediated autophagy (Zhang X. et al., 2018). Macroautophagy is the most widely studied form of autophagy. Nonetheless, over 30 autophagy-related genes (Atgs) and proteins have been found to participate in the process of autophagy (Díaz et al., 2017).

Presently, two classic signaling pathways from the entire autophagy signaling network have been described to inhibit or promote cell autophagy (Inoki et al., 2012; Shao et al., 2016). Type I PI3K-mammalian target of rapamycin (mTOR) signaling pathway is a classic inhibitory pathway. It is triggered under nutrient-rich conditions and stimulates mTOR and mTOR complex (MTORC1) via the protein kinase-B (also known as Akt) pathway, which then inhibits the formation of Atg1 complex (Kaur and Sharma, 2017). Another classic autophagy signaling pathway is induced by AMP-activated protein kinase (AMPK). AMPK is a sensor of stress and nutrient input. It activates ULK1 by inactivating mTORC1 or phosphorylating ULK1 at different residue serine kinase complexes to promote the autophagy process (Zhao et al., 2018). A study by Sciarretta et al. (2018) showed that autophagy plays a crucial role in inhibiting the occurrence and development of cardiovascular diseases such as MI, heart failure, and atherosclerosis. On the other hand, over-induction of the autophagy process may cause adverse effects to cells, the so-called “autophagy-dependent cell death” in the organism, which indicates the importance of controlling the degree of autophagy induction in disease treatment (Lambelet et al., 2018; Wang P. et al., 2018). Thus, autophagy seems to be a double-edged sword in the treatment of MI. Baseline autophagy can limit the death of cardiomyocytes, while excessive autophagy can aggravate the damage of cardiomyocytes. Demircan et al. (2018) also reported that patients with coronary heart disease or acute MI had an over-regulation of autophagy than the healthy controls. However, decreased autophagy and mitochondrial damage may lead to a weakened host’s response to hypoxic-ischemic injury, thereby adversely affecting cardiomyocytes (Ham and Raju, 2017). Laboratory research on rat models showed that autophagy could reduce the scope of acute MI after ligation of the left anterior descending artery (Aisa et al., 2017). Studies have shown that the basic autophagy process in cardiomyocytes is upregulated via the AMPK-mTOR signaling pathway, leading to reduced MI in animal models of acute MI (Foglio et al., 2017). As mentioned above, baseline autophagy or appropriately induced autophagy protects cardiomyocyte ATP production by maintaining cell homeostasis, degrading organelles or misfolded proteins, thereby protecting against ischemic injury. However, it is reported that under severe ischemia, excessive autophagy of the heart promotes cell death and worsens heart function (Li et al., 2018; Liu C. Y. et al., 2018). Interestingly, mitochondrial aldehyde dehydrogenase 2 (ALDH2) is an enzyme known to catalyze aldehyde oxidation. It can significantly promote the autophagy process during ischemia by activating AMPK and down-regulating mTOR, thereby producing cardioprotection. On the contrary, during reperfusion, ALDH2 can inhibit autophagy by activating Akt and mTOR, thereby protecting cardiomyocytes from cell death caused by hypoxia and reoxygenation (Ma et al., 2011). In summary, these studies all show that severe ischemia-induced excessive autophagy could aggravate acute MI.

## Pyroptosis

Pyroptosis is a regulated form of cell death closely associated with innate immune responses, characterized by plasma membrane permeability and the extracellular release of inflammatory cytokines and rupture of the plasma membrane mediated by gasdermin-D (GSDMD) (Shi et al., 2017). Regards morphology, cell pyroptosis, combines the characteristics of necrosis and apoptosis, including the formation of necrotic cell membrane pores, cell swelling, and membrane rupture, resulting in cytoplasmic content leakage nuclear condensation, and DNA fragmentation during apoptosis. In contrast to apoptosis, pyroptosis does not involve releasing cytochromes, and the mitochondrial integrity is maintained (Fink and Cookson, 2006; Cervantes et al., 2008). In a typical pyrophosphate signaling pathway, pathogen-related molecular patterns (PAMPs) or risk-related molecular patterns (DAMPs) are detected by different inflammasomes, which are composed of nucleotide-binding oligomerization domain (NOD), NOD-like receptors (NLR) family (NLRP3, NLRP1, NLRC4, NLRP9, and NLRP6), PYHIN protein family, and pyrin protein composition. These activated inflammasomes trigger the activation of caspase-1, which eventually leads to pyroptosis (Heilig and Broz, 2018). To trigger pyrolysis, the signal domains of NLR, AIM2, and pyrin (such as PYD) bind to ASC, while ASC recruits and activates pro-caspase-1, producing active caspase-1 (Aachoui et al., 2013). Activated caspase-1 not only processes and maturates IL-1β/18 but also cleaves the GSDMD intermediate junction, releasing intramolecular inhibition of the gasdermin-N domain and inducing cell pyroptosis by forming a 10–15 nm diameter pore on the cell membrane (Shi et al., 2015; Ding et al., 2016).

In the atypical pyroptosis pathway, caspase-11 and caspase-4/5 are primarily involved and activated by cytoplasmic lipopolysaccharide (LPS), where the CARD domain recognizes the lipid part of LPS (Yang J. et al., 2015; Abu Khweek and Amer, 2020). Activated caspase-4/5/11 directly triggers pyrolysis and the release of IL-1α and HMGB1 after cutting the GSDMD-induced membrane pore formation and subsequent cell membrane rupture. Activated caspase-4/5/11 also indirectly processes IL-1β through the atypical NLRP 3/ASC/caspase-1 pathway, which is formed by GSDMD pore formation/K + efflux or through vascular wall protein-1 cleavage/ATP release/P2 × 7/K + release mediated (Kayagaki et al., 2015; Rühl and Broz, 2015).

MI is associated with a sterile inflammatory response that leads to white blood cell aggregation (Takahashi, 2010). The white blood cells that accumulate at the MI site, including neutrophils and macrophages, release inflammatory cytokines, chemokines, and proteases, further aggravating the inflammatory response after MI, promoting myocardial injury, and remodeling. This sterile inflammatory response may be mediated by Toll-like receptors (TLRs) and NLRs. NLR is an integral part of the inflammasome that mediates the release of IL-1β (Shi et al., 2015). Studies have shown that the NLRP3 inflammasome plays a vital role in MI (Toldo and Abbate, 2018). During MI, activation of NLRP3 is associated with the leakage of lysosomal cathepsin-B, induction of K + outflow, ROS production, and other mediators (Liu et al., 2017). Inflammatory bodies were observed in a mouse model of acute MI, with increased ASC, NLRP3, and Caspase-1 in scar tissue and the cytoplasm of adjacent myocardial cells in the infarction area (Mezzaroma et al., 2011). Besides, oxidative stress induced by MI can also cause cell pyroptosis. The inhibition of oxidative stress can reduce pyroptosis and decrease the activities of NF-κB and GSDMD. Inhibition of NF-κB also inhibits oxidative stress-regulated pyrogen death by reducing GSDMD (Lei et al., 2018). These results suggest that myocardial cell damage during MI is partly related to pyroptosis.

## Ferroptosis

Ferroptosis is a form of regulatory cell death caused by iron-dependent lipid peroxidation (Stockwell et al., 2017). Among the members of the GPX family, Gpx4 is believed to specifically catalyze the reduction of lipid peroxides, thereby scavenging lipid reactive oxygen species (Stockwell et al., 2017). The absence of Gpx4 causes lipid peroxides and lipid ROS accumulation and in a 12/15-lipoxygenase (12/15LOX) dependent manner, leading to cell death (Seiler et al., 2008). On the other hand, the reduction of lipid peroxides by GPX4 requires the oxidation of glutathione. Glutathione is primarily synthesized by cysteine and inhibits the cystine-glutamate transporter (xCT), or cysteine deprivation will cause glutathione depletion, which will induce ferroptosis (Jiang et al., 2015). The recently discovered iron death inhibitor protein-1 (FSP1) (previously known as apoptosis-inducing factor mitochondria-2, AIFM2), an influential ferroptosis resistance factor, is believed to be a vital component of a non-mitochondrial CoQ antioxidant system, which is parallel to the typical glutathione-based GPX4 pathway anti-ferroptosis system.

## Exosomes Reduce Cardiomyocyte Death After Myocardiac Injury

**FIGURE 1:**
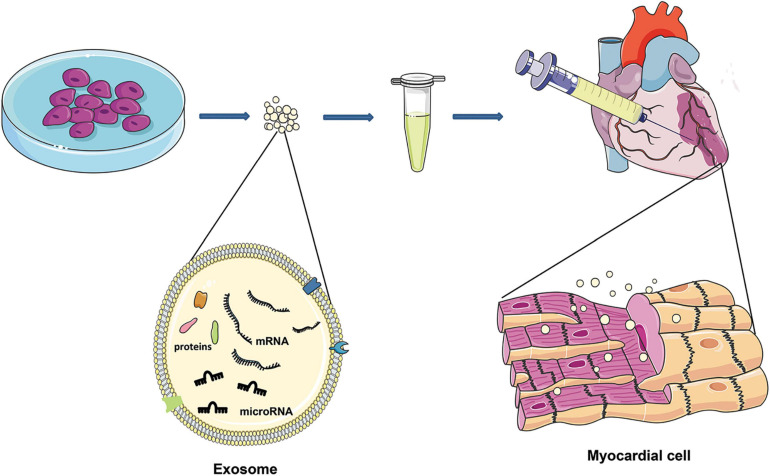
After MI, exosomes of different cell sources are injected around the infarction site, and exosomes can enter the damaged cardiomyocytes to reduce cardiomyocyte death. Exosomes are characterized by lipid bilayers, transmembrane and non-membrane binding proteins, and a high concentration of nucleic acids (DNA, mRNA, microRNA, and lncRNA).

Recent studies have shown that the myocardial protective effect of the injected stem cells is not produced by direct differentiation itself but mediated by exosomes secreted from stem cells (Kishore and Khan, 2016; Jung et al., 2017). Thus, exosomes may be an effective alteration to overcome the shortcomings of cell therapy. Exosomes with significant cargos may merge their membrane contents into the recipient cell membrane and transport complex signal molecules into the recipient cell (Valadi et al., 2007). Exosomes play an essential role in cell-to-cell communication, regulating various cellular processes in target cells, including adjacent cells and cells in remote parts (Stoorvogel, 2012). Several studies have reported that stem cell-derived exosomes could be used to treat ischemic disease (Lai et al., 2010; Arslan et al., 2013; Ibrahim et al., 2014; Figure 1).

Besides, exosome therapy has advantages where tumorigenesis and embolism caused by cell transplantation are significantly lower when using exosomes because they are much smaller than cells (Park K. S. et al., 2019). Also, the membrane of exosomes is primarily a lipid bilayer with fewer binding proteins. Thus, their immunogenicity is lower than that of cells and could reduce the recognition and phagocytosis of immune cells (Hood and Wickline, 2012). Moreover, with exosomes, there is (i) no self-metabolism and changes in the body temperature will not affect its biological activity where it could better exert its functional characteristics (Lai et al., 2013) and (ii) exosomes are lipid-bound nano-scale vesicles that can freely pass through the blood-brain barrier (Konala et al., 2016). Furthermore, due to their stable nature, they are thus, (i) suitable for storage and transportation as exosomes can be stored at −80°C for 4 weeks without losing their physical properties (Agrawal et al., 2017). Finally, the double-layer lipid membrane of the exosome can encapsulate and protect its contents, hence preventing the rapid degradation of cytokines and RNA and deliver therapeutic agents to the target site (Didiot et al., 2016). Generally speaking, molecular carriers with biological activity in exosomes include lipids, proteins, and nucleic acids, such as DNA, mRNA, miRNA, and lncRNA. Though exosomes have these advantages and have great potential in treating cardiovascular diseases, there are also some problems in practice. For example, the extraction of exosomes is complicated and time-consuming, and uneven purification can cause significant differences in therapeutic effects (Kishore and Khan, 2017). Potential therapeutic advances of exosomes include their role in promoting angiogenesis, anti-apoptosis, anti-immunogenicity, proliferation, and anti-fibrosis (Chaput and Théry, 2011; Wang Y. et al., 2015; Yang, 2018; Ye et al., 2019).

It is essential to note that MI and I/R injury could lead to myocardial injury. Various types of cell-derived exosomes play a crucial role in repairing cell damage after myocardial tissue injury. Here we describe several programmed death of cardiomyocytes after MI and I/R injury and the regulatory mechanisms of exosomes.

### Exosomes Reduce Apoptosis

The mechanism of exosomes from different cell sources about the anti-apoptosis of cardiomyocytes was summarized in Table 1. Bone marrow mesenchymal stem cell (BMMSC)-derived exosomes play a key role in repairing myocardial injury caused by tissue reperfusion, and the exosomal miR-486-5p inhibit myocardial apoptosis by PTEN de-activation and through PI3K/Akt pathway (Sun et al., 2019). In addition, a variety of pretreatments with BMMSCs can change the composition of the secreted exosomes. In which the most common used method is hypoxic pretreatment. Studies have found that with hypoxia pretreatment of BMMSCs, the miR-214 in secreted exosomes is significantly increased, and further confirmed that the exosomal miR-214 is transferred to cardiomyocytes and inhibit the expression of CaMKII (Wang Y. et al., 2018). Another study showed that hypoxic pretreatment of mouse BMMSCs increased the expression of miR-125b-5p in their exosomes. After injected into the infarcted area, the ability of cardiomyocytes to resist apoptosis were significantly enhanced through the inhibition of p53 and BAK1 (Zhu et al., 2018). Furthermore, the increased expression of miR-24 and miR-210 in the exosomes of hypoxic BMMSCs can also increase the anti-apoptotic potency of cardiomyocytes. The specific anti-apoptotic mechanism has not been elucidated, but the expression of exosomal miR-210 depends on the amount of neutral sphingomyelinase 2 (nSMase2). It is believed that gene or microRNA modified exosomes may convert the ability of these exosomes to resist cardiomyocyte apoptosis. Studies have found that GATA binding protein-4 (GATA-4) regulates the expression of miR-15 family members in BMMSCs and improves their survival rate in an ischemic environment (Yu et al., 2013). Other methods including miR-125b overexpression in exosomes via transfecting miR-125b into BMMSCs to achieve the effect of anti-cardiomyocyte apoptosis (Chen et al., 2020). The target of miR-125b is SIRT7, which is involved in the regulation of cardiac cell apoptosis (Araki et al., 2015). All of the above are the effects of exosomes derived from BMMSCs against cardiomyocyte apoptosis after ischemia.

**TABLE 1:** Mechanisms of exosomes from different cell sources against cardiomyocyte apoptosis.

Derivation of exosome	Stimulus	Molecular mediator(s)	Mechanisms	Biological effects	References
BMMSCs	−	miR-486-5p	miR-486-5p/PTEN/PI3K/AKT	Apoptosis↓	Sun et al., 2019
BMMSCs	Hypoxia	miR-214	miR-214/CaMk2	Apoptosis↓, oxidative stress↓	Wang Y. et al., 2018
BMMSCs	Hypoxia	miR-125b-5p	miR-125b-5p/p53andBAK1	Apoptosis↓	Zhu et al., 2018
BMMSCs	Hypoxia	miR-210and miR-24	Not investigated	Apoptosis↓	Zhu et al., 2018
BMMSCs	Transduction with GATA-4	miR-19a	miR-19a/PTEN/AKT miR-19a/BIM	Apoptosis↓	Yu et al., 2015
BMMSCs	Transduction with SDF1	SDF1	SDF1/PI3K/mTOM	Apoptosis↓, microvascular regeneration	Gong et al., 2019
BMMSCs	Transduction with miR-125b	miR-125b	miR-125b/SIRT7	Apoptosis↓, inflammatory factor↓	Chen et al., 2020
ADMSCs	−	Not investigated	S1P/SK1/S1PR1	Apoptosis↓, fibrosis↓, M2 macrophages polarization	Deng et al., 2019
ADMSCs	−	miR-214	miR-214/Bcl2L11 miR-214/SLC8a1	Apoptosis↓	Eguchi et al., 2019
ADMSCs	Transduction with miR-146a	miR-146a	miR-146a/EGR1/TLR4/NFκB	Apoptosis↓	Pan et al., 2019
hucMSCs	−	miR-19a	miR-19a/SOX6/AKT/JNK3/Caspase3	Apoptosis↓	Huang et al., 2020
hucMSCs	Transduction with TIMP2	Not investigated	AKT/sfrp2	Apoptosis↓, oxidative stress↓angiogenesis?	Ni et al., 2019
IPSCs	−	miR-21 miR-210	Not investigated	Apoptosis↓	Adamiak et al., 2018

Exosomes derived from other stem cells have also been extensively studied. Studies have found that exosomes derived from adipose-derived mesenchymal stem cells (ADMSCs) can regulate S1P/SK1/S1PR1 signals (Deng et al., 2019). Because miR-214 is highly expressed in exosomes derived from ADMSCs, they could inhibits the expression of Bcl2L11 and SLC8a1 after delivered into the myocardiac infarcted site (Eguchi et al., 2019). The protein encoded by Bcl2L11 may induces apoptosis through bax activation or anti-apoptotic proteins neutralization (Shukla et al., 2017). The sodium/calcium exchange protein encoded by SLC8A1 causes cardiomyocyte calcium overload-related apoptosis under cardiac stress (Aurora et al., 2012). Studies have showed the anti-apoptotic ability of hypoxic cardiomyocytes by overexpressing miR-146a in ADMSCs derived exosomes. The anti-apoptotic effect of miR-146a mainly caused by the inhibition of early growth response factor 1 (EGR1) in I/R injured tissue (Yang L. et al., 2015; Pan et al., 2019). Overexpressing miR-19 in exosomes derived from umbilical cord MSCs could regulate the AKT/JNK3/caspase-3 axis via inhibiting the expression of SOX6, which may lead to the decrease of hypoxic cardiomyocyte apoptosis (Huang et al., 2020). Exosomes derived from TIMP2 overexpressed umbilical cord MSCs significantly enhance the anti-apoptotic ability of hypoxic cardiomyocytes via the inhibition of Bax and pro-caspase expression (Ni et al., 2019). Exosomes derived from iPSCs also play a key role in resisting hypoxic myocardial apoptosis. Studies have shown that exosomal miR-21 and miR-210 may be key factors in anti-cardiomyocyte apoptosis (Wang Y. et al., 2015; Adamiak et al., 2018). Previous studies reported that miR-21 inhibits apoptosis by targeting PDCD4/AP-1 in infarcted cardiomyocytes (Xiao et al., 2016). Interestingly, a study showed that exercise can rapidly increase the exosomes in plasma, which could effectively resist cardiomyocyte apoptosis via activating ERK1/2 and HSP27 in hypoxia-reoxygenated cells (Bei et al., 2017).

### Exosomes Regulate Autophagy-Dependent Cell Death

**FIGURE 2:**
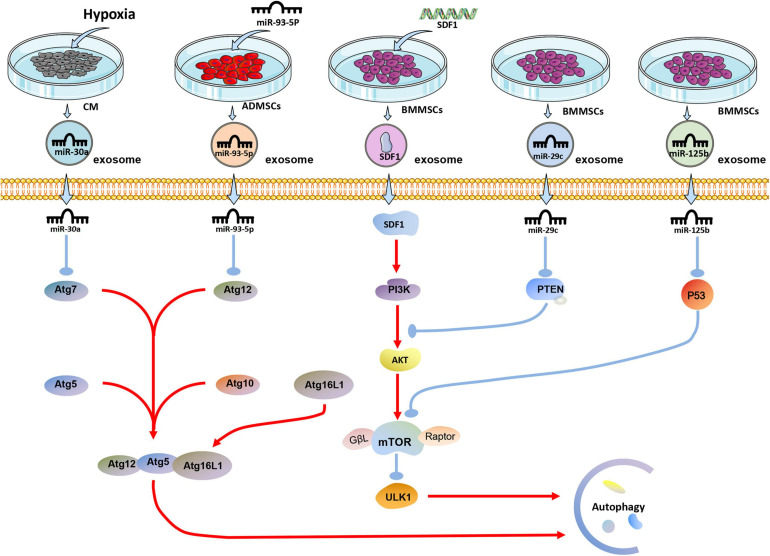
The effect of exosomes derived from differently treated cells on autophagy of cardiomyocytes after MI. The miR-30a in exosomes secreted by cardiomyocytes after hypoxia is targeted to Atg7 for anti-autophagy; miR-93-5P in exosomes secreted by ADMSCs transfected with miR-93-5P targeted Atg12 for anti-autophagy; SDF1 protein secreted by BMMSCs transfected with SDF gene activates PI3K/AKT pathway to resist autophagy; miR-29c and miR-125b in exosomes derived from BMMSCs target PTEN and P53, respectively, to resist autophagy.

Studies have shown that exosomes can reduce myocardial autophagy and death during MI or I/R injury (Figure 2). In 2018, it was discovered that exosomes derived from human MSCs reduce I/R injury by inhibiting cardiomyocyte autophagy, but the specific mechanism has not been proved (Jiang et al., 2018). Experiments have shown that miR-125b-5p is highly expressed in exosomes derived from BMMSCs. Exosomal miR-125b-5p inhibits the expression of P53 and reduces the autophagy of myocardial cells in the infarcted site when delivered to the MI heart (Xiao et al., 2018). In the mouse myocardial I/R injury model, the expression of miR-29c decreased, and the autophagy flow of myocardial cells increased. The high expression of miR-29c can reduce the excessive autophagy of hypoxic myocardium by directly inhibiting the expression of PTEN, thus inhibiting the I/R-induced excessive autophagy via the PTEN/AKT/mTOR signaling pathway (Li et al., 2020). Besides, through transfection, over expression of miR-301 in BMMSCs increases the expression of exosomal miR-301, which significantly reduces the ratio of LC3-II/LC3-I and increases P62 relative expression in the infarcted myocardial tissue. Notably, the ratio of LC3-II/LC3-I increased, and the expression level of P62 decreased when the degree of autophagy was enhanced (Li Y. et al., 2019).

Similarly, by overexpressing SDF1 in BMMSCs, the expression of SDF1 in exosomes was significantly increased, which significantly increased the expression of Bcl-2 in hypoxic cardiomyocytes. On the other hand, Bax, Beclin-1, LC3, and LC3II/LC3I ratio were significantly reduced, reducing excessive autophagy in cardiomyocytes (Gong et al., 2019). Studies have shown that ADMSCs overexpressing miR-93-5p can effective package miR-93-5p into exosomes. The exosomes would essentially deliver exosomal miR-93-5p to the site of MI to inhibit excessive autophagy. *In vitro* experiments show that miR-93-5p achieves these results by targeting Atg7 (Liu J. et al., 2018). Another interesting study found that miR-30a is highly enriched in exosomes in the serum of patients with acute MI. Exosomes are an important communication route among hypoxic cardiomyocytes, and exosomal miR-30a may inhibit autophagy in hypoxic cardiomyocytes by inhibiting Beclin-1 and Atg12 (Yang Y. et al., 2016).

### Exosomes Reduce Pyroptosis

In the process of MI, NLRP3 is one of the critical molecules for myocardial cell pyroptosis. Studies have shown that exosomes derived from human MSCs can significantly reduce the expression of NLRP3 and Caspase-1 in the I/R myocardium, then reduce the myocardial pyroptosis. It has been proven that miR-320b in exosomes played a crucial role in myocardial cell pyroptosis, with NLRP3 being the target gene of miR-320b (Tang et al., 2020). Macrophage-derived exosomes occupy a large part of the circulating microcapsules in the blood (McDonald et al., 2014). Studies have found that exosomes derived from M2 macrophages can reduce myocardial damage caused by I/R injury via the high expression of exosomal miR-148a and through TXNIP-NLRP3-caspase-1 path way (McDonald et al., 2014). SIRT1 plays a central role in regulating various cellular processes related to heart development and cardiovascular diseases (Chen et al., 2010). SIRT1 expression is down-regulated in MI, and overexpression of SIRT1 can effectively reduce myocardial damage caused by MI (Mori et al., 2014).

**FIGURE 3:**
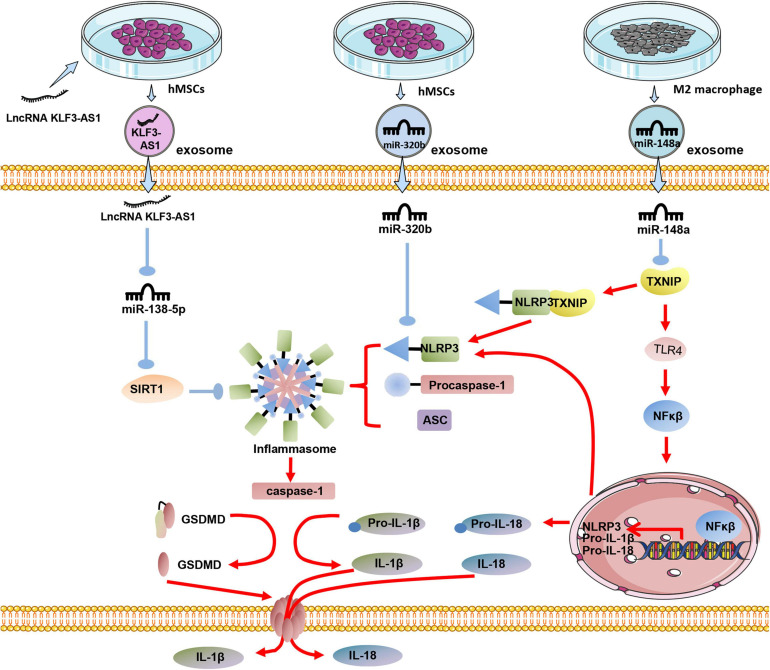
The effect of exosomes derived from differently treated cells on the pyrolysis of cardiomyocytes after MI. Human MSCs are transfected with LncRNA KLF3-AS1, and the secreted exosomal LncRNA KLF3-AS1 are highly expressed. LncRNA KLF3-AS1 inhibits miR-138-5p then inhibits pyroptosis; Exosomal miR-320b derived from unmodified human MSCs targets NLRP3 to inhibit cardiomyocyte pyroptosis; Exosomal miR-148a in exosomes derived from M2 macrophages inhibits TXINP expression to protect against cardiomyocyte pyroptosis.

Overexpressing LncRNA KLF3-AS1 in BMMSCs might increase the expression of LncRNA KLF3-AS1 in exosomes. LncRNA KLF3-AS1 acts as a sponge of miR-138-5p in ischemic cardiomyocytes, and the absorption of miR-138-5p increases the expression of SIRT1 in cardiomyocytes. Besides the inhibition of Caspase-1, inflammatory cytokine IL-1β, SIRT1 can also inhibit the expression of NLRP3 and Asc, thus reducing the pyroptosis of cardiomyocytes (Mao et al., 2019; Figure 3).

### Exosomes Reduce Ferroptosis

In a mouse model of cardiac I/R injury, both the iron chelator desferrioxamine (DFO) and the glutamine decomposition inhibitor compound-968 inhibit ferroptosis, reduce the size of MI, and improve heart function (Gao et al., 2015). Another study showed that treatment with Ferrostatin-1 or the iron chelator dexrazoxane in mice reduced the size of infarction and serum markers of myocardial injury during I/R (Fang et al., 2019). Proteomics showed that the protein level of GPX4 in myocardial cells decreased the first day and 1 week after MI in mice. The GPX4, however, slightly increased 8 weeks after infarction. RNA-seq and qRT-PCR analysis showed that the down-regulation of GPX4 occurred at the transcription level, and the down-regulation of GPX4 would cause ferroptosis of cardiomyocytes during MI (Park T. J. et al., 2019).

**FIGURE 4:**
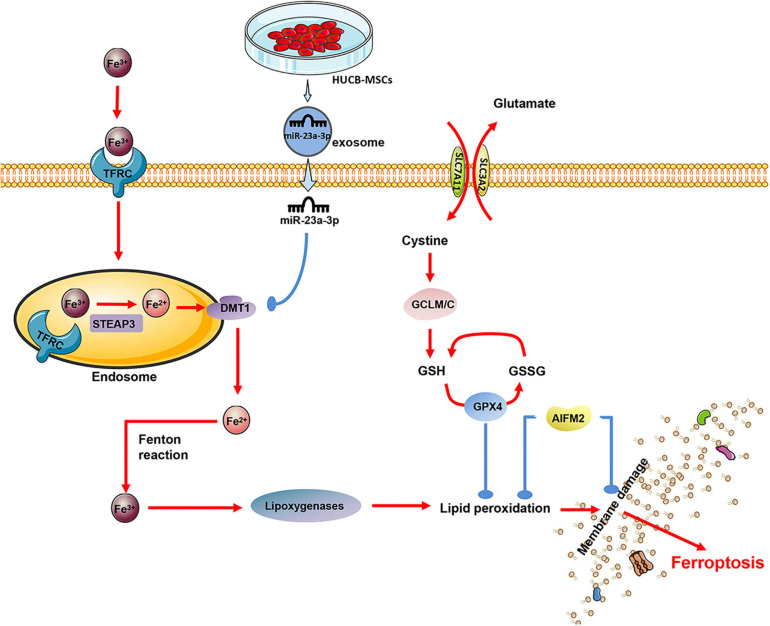
The exosomes derived from human umbilical cord blood (MSCs) contain a significant amount of miR-23a-3p, which inhibits the expression of DMT1 after entering the cardiomyocytes, thereby reducing intracellular lipid oxidation and inhibiting the ferroptosis of cardiomyocytes.

Significantly, Fe3^+^ is introduced via the transferrin receptor (TR). In the endosome, Fe3 + is first converted to Fe2^+^ via the Metallo-reductase six-transmembrane epithelial antigen of prostate 3 (Steap3) and later released from the endosome via the divalent metal transporter-1 (DMT1) (Xue et al., 2016; Pujol-Giménez et al., 2017). It is reported that the ferroptosis of cells is related to the up-regulation of DMT1 (Li L. B. et al., 2019; Yu et al., 2019). Notably, the expression of DMT1 in acute MI mice at 24 and 48-h was significantly higher than that in the sham operation group, while miR-23a-3p was highly expressed in exosomes derived from human MSCs (Ferguson et al., 2018). By transplanting exosomes, miR-23a-3p suppresses the iron death of myocardial cells by targeting DMT1 and improving MI’s cardiac function (Song et al., 2020; Figure 4).

## Conclusion

In the review herein, we discussed the anti-cell death potential of various exosomes from different cells, primarily stem cells for heart injury caused by MI or I/R. The above research shows that the same cell undergoes different treatments to change the type and proportion of the content in the exosomes from which it originates. Significantly after hypoxic treatment of stem cells, the protective substances in their exosomes increase. Studies have found that after LPS pretreated stem cells, their exosomes can enhance the M1 polarization of macrophages (Kang et al., 2018). All these indicate that modified and/or unmodified stem cells can increase the production of protective substances when they perceive danger signals, and exosomes serve as media to deliver protective substances. For the miRNAs and proteins, we believe can play a protective role, the cells are overexpressed by transfection, and these substances can also be delivered through exosomes as a medium to achieve cytoprotective effects. After modification of non-stem cells, can these cell-derived exosomes achieve the same protective effect? We believe that MSC and other stem cell-derived exosomes may be superior to other cells, because a large number of experiments have shown that stem cell-derived exosomes can play a protective role in cells, and the enhancement of protective factors can improve their ability. Besides, stem cell-derived exosomal microRNAs are potent regulators of cardiomyocytes’ survival and functional properties, cardiomyocyte progenitor cells, and endothelial cells. The exocytosis of other cells including exosomes derived from cardiac lineage-committed cells (cardiospheres, cardiac cells differentiated from pluripotent stem cells) is also worthy of attention. The exosomes naturally produced by cells have been proven to produce the desired effect. Thus, the research for engineered exosomes that can produce potent effects to optimize cardioprotection should be the next step of action. The purpose of the modification is to produce exosomes that encapsulate the desired molecule. Future research in this area should focus on identifying the role of specific molecules in exosomes. Also, exploring the mechanism of the exosome loading process will be a valuable addition. Simultaneously, it is also necessary to consider that different microRNA target combinations should be tested in different cell types and different cardiovascular environments while paying attention to safety and effectiveness.

Despite the composition of exosomal contents, keen attention should be paid to saving cardiomyocytes, particularly those involved in multiple programmed deaths. For instance, p53 participates in apoptosis and autophagy death after MI, as one of the most common natural stresses of p53 activation is hypoxia. Under hypoxic conditions, neonatal rat cardiomyocytes may lead to intranuclear cleavage of genomic DNA, accompanied by increased p53 trans-activation activity and p53 protein accumulation (Kirshenbaum and de Moissac, 1997). The heterotopic expression of the anti-apoptotic gene Bcl-2 is sufficient to antagonize p53 induced apoptosis (Ng et al., 1999). Over-activation of p53-myocardin signaling in autophagy during myocardial infarction leads to the death of myocardial cells and accelerated ischemic injury (Liu C. Y. et al., 2018). The overexpression of p53 after MI may enhance apoptosis and autophagy of cardiomyocytes, and the inhibition of this target can achieve double the result with half the effort. Therefore, for the increase of protective substances in exosomes, the typical target of cardiomyocyte hypoxia death should be significantly considered to achieve maximum effectiveness.

## Author Contributions

XW and CF carried out the data collection and assembly of data, data analysis, and wrote the manuscript. XW, JP, and JG carried out the data collection and assembly. XW, CI, and JY carried out data analysis and interpretation, and manuscript revising. CF carried out the conception and design, and manuscript revising. All authors read and approved the final manuscript.

## Conflict of Interest

JG was employed by the company Hunan Fangsheng Pharmaceutical Co. Ltd. The remaining authors declare that the research was conducted in the absence of any commercial or financial relationships that could be construed as a potential conflict of interest.

## Abbreviations

3 ′ UTR: 3 ′ untranslated region
ALDH2: aldehyde dehydrogenase 2
AMPK: AMP-activated protein kinase
Atgs: autophagy-related genes
CVD: Cardiovascular disease
DFO: desferrioxamine
DMT1: divalent metal transporter 1
EGR1: early growth response factor 1
ESCRT: endosomal sorting complex
GATA-4: GATA binding protein-4
GSDMD: gasdermin D
HSPs: heat shock proteins
I/R: ischemic-reperfusion
LPS: lipopolysaccharide
MI: myocardial infarction
mTOR: mammalian target of rapamycin
nSMase2: neutral sphingomyelinase 2
PAMPs: athogen-related molecular patterns
SDF1: stromal-derived factor 1
Steap3: six-transmembrane epithelial antigen of prostate 3
TIMP2: Tissue inhibitor of matrix metalloproteinase 2
NLR: nucleotide-binding oligomerization domain (NOD)-like receptor
TNF- α: tumor necrosis factor- α
TLRs: Toll-like receptors
TXNIP: thioredoxin-interacting protein.
